# MiR-124 Promote Neurogenic Transdifferentiation of Adipose Derived Mesenchymal Stromal Cells Partly through RhoA/ROCK1, but Not ROCK2 Signaling Pathway

**DOI:** 10.1371/journal.pone.0146646

**Published:** 2016-01-08

**Authors:** Ye Wang, Desheng Wang, Dawen Guo

**Affiliations:** 1 Department of Neurology, First Affiliated Hospital of Harbin Medical University, Harbin, Heilongjiang, 150001, China; 2 Department of Clinical Laboratory, First Affiliated Hospital of Harbin Medical University, Harbin, Heilongjiang, 150001, China; French Blood Institute, FRANCE

## Abstract

**Objective:**

Some recent studies suggest that multiple miRNAs might regulate neurogenic transdifferentiation of mesenchymal stromal cells (MSCs). In the present study, we hypothesized that the miR-124 can repress the expression of RhoA upon the neurogenesis of adipose derived MSCs (ADMSCs).

**Methods:**

MiRNA expression dynamics during neurogenic transdifferentiation of ADMSCs were measured. The expression of neuron-specific enolase (NSE), Tuj-1 (Neuron-specific class III beta-tubulin) and glial fibrillary acidic protein (GFAP), as well as electrophysiological properties, were detected after neurogenic transdifferentiation. The targeting of miR-124 over RhoA was verified by dual luciferase assay, qRT-PCR and western blot. The functions of miR-124 and the RhoA/ROCK signaling pathway were studied using gain and loss of function experiments *in vitro*.

**Results:**

MiR-124 is significantly upregulated during neurogenic transdifferentiation of ADMSCs. Knockdown of endogenous miR-124 hampered neurogenic transdifferentiation and the acquired electrophysiological properties. MiR-124 could directly target RHOA mRNA and repress its expression, through which it increased the proportion of transdifferentiated (transdiff.) cells with positive NSE, Tuj-1 and GFAP. RhoA/ROCK1, but not ROCK2 is a downstream signaling pathway of miR-124 in the process of transdifferentiation.

**Conclusion:**

MiR-124 is an important miRNA modulating neurogenic transdifferentiation of ADMSCs at least partly via the miR-124/RhoA/ROCK1 signaling pathway. These findings provided some fundamental information for future use of ADMSCs as an agent for regenerative medicine and cell therapy for neurological diseases.

## Introduction

Mesenchymal stromal cells (MSCs) are multipotent mesoderm derived adult stem cells that exist in the stroma of multiple tissues, such as bone marrow, umbilical cord blood, peripheral blood, adipose tissue, muscle, dermis and periosteum [1]. Since MSCs can transdifferentiate into osteogenic, chondrogenic, adipogenic, myogenic, fibroblastic, and neuronal lineages, they are considered as a promising source of cellular therapy and regenerative medicine [2,3]. In particular, human adipose-derived mesenchymal stromal cells (ADMSCs) are an attractive MSC source due to the minimally invasive accessibility and large availability [4]. However, the mechanisms underlying the neurogenic transdifferentiation of ADMSCs remain elusive.

MicroRNAs (miRNAs) are a class of small noncoding RNAs involved in posttranscriptional regulation of gene expression usually by binding to the 3’ untranslated region (UTR) of mRNAs [5]. Some recent studies indicate that multiple miRNAs might regulate neuronal-like transdifferentiation of MSCs. For example, miR-29a can modulate neuronal transdifferentiation of MSCs through targeting REST [6]. MiR-124 also can regulate neuronal-like transdifferentiation of MSCs by reducing specificity protein 1 (Sp1) expression [7]. In fact, miR-124 is a miRNA, of which the expression is progressively increased during development of central nervous system [8–10]. This suggests that miR-124 might be an important miRNA with unique functions during neuronal differentiation. Ectopic expression of miR-124 in mouse neurogenic stem cells induced morphological changes and marker expressions consistent with neuronal differentiation [11]. MiR-124 can promote neurite outgrowth in mouse P19 cells [12]. However, its downregulation is associated with reduced neurite outgrowth and the amount of acetylated a-tubulin [12]. Therefore, miR-124 might play an important role to maintain neuronal state of the cells. Since one miRNA usually has multiple targets and can regulate several signaling pathways simultaneously, it is necessary to further explore its downstream regulation.

Ras homolog gene family, member A (RhoA) is a small GTPase protein that is involved in regulation of the actin cytoskeleton in the formation of stress fibers [13]. Some recent studies revealed that RhoA and its downstream signaling pathways are involved in neuronal-like transdifferentiation of MSCs. Y-27632, a Rho-kinase (ROCK) inhibitor can promote MSCs transdifferentiation into neurons [14, 15]. Cyclic tensile loading, which is a mechanic method utilized to promote neuronal transdifferentiation of MSCs is also partly through the regulation of Rho GTPases activity [16]. Considering the importance of this signaling pathway, we decided to explore its upstream regulation in neurogenic transdifferentiation of ADMSCs. In the present study, we hypothesized that the miR-124 can repress the expression of RhoA upon the neurogenesis of ADMSCs.

## Materials and Methods

### Cell culture

This study was approved by the ethic committee of the first Affiliated Hospital of Harbin Medical University. Human ADMSCs were purchased from Invitrogene (R7788-110, Carlsbad, CA, USA) and cultured using the prepackaged MesenPRO RS^™^ Medium in an incubator with a humidified atmosphere and 5% CO_2_ at 37°C. Passage 3 ADMSCs were used for induction of transdifferentiation. One previous study suggest that sphere formation culture models have better survival time and increased expression of neuronal markers than adherent models [17]. However, the sphere formation culture models may also has a drawback since only around 8% of cells within the MSCs’ population were able to generate neurospheres when cultured in neurogenic stem cell (NSC) culture conditions [3]. In this study, ADMSCs in some groups were transfected with Rho shRNA, ROCK1 shRNA or ROCK2 shRNA lentiviral particles which might affected the sphere formation and transdifferentiation [4, 5], so we just used the adherent model which was widely used in previous studies [1, 6, 7].

Induction of neurogenic transdifferentiation of the naive ADMSCs and cells with miR-124, RhoA, ROCK1 or ROCK2 knockdown followed a two-step protocol introduced in one previous study [7]. Briefly, 5×10^4^ ADMSCs were cultured in 24-well plates per well. At 30% confluence, the cell culture was supplemented with 5μg/ml insulin, 5 ng/ml bFGF-2, 0.5 μM retinoic acid and 15 ng/ml EGF for 5 days. Then, the culture medium was changed to the Step-2 induction medium supplemented with 100 μM cAMP, 500 μM 3-isobutyl-1-methylxanthine (IBMX); 100 ng/ml nerve growth factor (NGF) and 10 μM forskolin for another 5 days. The morphological images of the cells before and after induction were captured using a microscope with a digital camera (Olympus IX-71, Olympus, Japan).

### Isolation and culturing of primary rat hippocampal neurons

Procedures involving animals were reviewed and approved by the Animal Care and Use Committee of the Harbin Medical University. All animal studies were performed in accordance with the ethical standards according to the Declaration of Helsinki. Sprague Dawley rats were purchased from the Center for Experimental Animals, Harbin Medical University. Six 18-day-old rats were euthanized with an overdose of thiopental by intraperitoneal injection. Primary rat hippocampal neurons were prepared from the brains according to the method introduced in a previous study [18]. Briefly, hippocampi were isolated from total brain and incubated with trypsin at 37°C. The dissociated hippocampal neurons were then plated at 24,000 cells/cm^2^ and grown in neurobasal medium supplemented with B27.

### Reagents and cell transfection

The pLV-miR-124 expression plasmid, the pLV-miR-124 locker plasmid and the lentiviral packaging vector mix were purchased from Biosettia (San Diego, CA, USA). The lentiviral pLV-miR expression vector or the pLV-miR locker vectors were produced by co-transfection with helper plasmids mixture into HEK 293T cells using Lipofectamin 2000 (Invitrogen) according to manufacturer’s instruction. The ready-to-use human RhoA shRNA lentiviral particles (sc-44209-V), ROCK1 shRNA lentiviral particles (sc-29473-V) and ROCK2 shRNA lentiviral particles (sc-29474-V) were purchased from Santa Cruz Biotech (Santa Cruz, CA, USA). To overexpress or suppress the genes, the ADMSCs were infected with corresponding lentiviral particles with the presence of 8μg/ml polybrene (Sigma-Aldrich, St Louis, MO, USA).

### Immunofluorescence staining

To assess neurogenic transdifferentiation, the cells were stained with neuron-specific enolase (NSE), Tuj-1 (Neuron-specific class III beta-tubulin) and glial fibrillary acidic protein (GFAP). Briefly, cells were fixed with 4% paraformaldehyde for 20 min. Then the cells were permeabilized using PBS containing 0.5% Triton-X 100 (Sigma Aldrich) for 1 hour and then blocked using PBS supplemented with 4% BSA and 0.3% Triton-X 100 for another hour. After these treatments, the cells were incubated primary anti-NSE antibody (1:500, ab53025, Abcam, Cambridge, MA, USA), anti-Tuj-1 (1:500, ab18207, Abcam) or anti-GFAP (1:200, ab48050, Abcam) overnight at 4°C. Immunolabeling was visualized using DyLight^®^ 594-conjugated Donkey anti-rabbit IgG polyclonal antibody (1: 1000, Ab96921, Abcam) for 2 h at room temperature in PBS. The nuclei were stained with 5 mg/ml DAPI for 15min and were examined on a fluorescent microscope. Digital Immunofluorescent images were obtained using an Olympus IX71 inverted microscope (Olympus, Tokyo, Japan).

### Microarray of miRNAs expression

ADMSCs before induction, after the first step of induction and after the second step induction were used for microarray of miRNAs expression. Briefly, total miRNAs in the cell samples were extracted using the miRVana miRNA Isolation Kit (Ambion, Austin, TX, USA) according to the manufacturer’s instructions. The miRNA samples were labeled using the miRCURY Hy3/Hy5 Power labeling kit (Exiqon, Vedbaek, Denmark) according to the manufacturer’s guidelines. The labeled miRNA samples were hybridized on the miRCURYTM LNA microRNA Array (v.14.0) (Exiqon) according to the array manual. Then, the microarrays were scanned with the Axon GenePix 4000B microarray scanner (Axon Instruments, Foster City, CA, USA). The scanned images were imported into GenePix Pro 6.0 software (Axon Instruments) for grid alignment and data extraction. After background subtraction and signal normalization, the miRNAs with at least 3-fold difference before and after induction were candidates for further analysis (p<0.05).

### QRT-PCR analysis

Total RNAs of the cells were extracted using Trizol Reagent (Invitrogen, Carlsbad, CA, USA) according to the manufacturer’s instruction. Then the RNA samples were used for reverse transcription to get the first strand cDNA by using the PrimeScript^®^ RT reagent kit (TaKaRa, Dalian, Liaoning, China). The expression of RhoA mRNA and neurogenic markers NSE, Tuj-1 and GFAP [10] were quantified using gene specific primers (S1 Table) and SYBR^®^ Green PCR Master Mix (Applied Biosystems, Foster City, CA, USA). GAPDH was used as the endogenous control gene. MiR-124 expression was detected using TaqMan MicroRNA Assay Kit (Applied Biosystems, Foster City, CA, USA), with U6 snRNA used as the endogenous control. All qRT-PCR analysis was performed using an ABI Prism 7500 (Applied Biosystems, Foster City, CA, USA). The results of qRT-PCR analysis were presented using 2^-ΔΔCT^ method.

### Western blot analysis of RhoA expression

Cells were lysed using a RIPA buffer (Beyotime, Shanghai, China) and the protein concentration was measured using a BCA Protein Assay Kit (Beyotime). The protein samples were separated by 10% SDS-PAGE and then transferred onto a PVDF membrane. After blocking with 5% nonfat dry milk, the membranes were incubated with primary anti-RhoA (1:1000, ab86297, Abcam) overnight at 4°C. Membranes were washed and incubated with HRP-labeled secondary antibodies (anti-rabbit IgG, 1:10000, AP188P, Merck Milipore). The band signals were visualized using the ECL Western blotting substrate (Promega, Madison, WI, USA).

### Dual luciferase assay

Chemically synthesized miR-124 mimics and the scramble negative controls were purchased from RiboBio (Shanghai, China). Putative binding sites between miR-124 and 3’UTR of RhoA were predicted using TargetScan 6.2. Wild type or mutant (without miR-124 binding site) human RhoA 3'UTR sequences with flanking *SacI* and *XhoI* restriction enzyme digestion sites were chemically synthesized. Then the wild type and mutant sequences were inserted between *SacI* and *XhoI* sites of pGL-3 promoter vector respectively. The recombinant plasmids were named as pGL3-RhoA-WT and pGL3-RhoA-MUT respectively. To assess the influence of putative miR-124 binding site on luciferase expression, HEK 293T cells were co-transfected with 200ng recombinant plasmids, 20ng phRL-TK plasmid carrying the Renilla luciferase gene and 50nM miR-124 mimics or negative control using Lipofectamin 2000 (Invitrogen). 24h after transfection, luciferase activity was analyzed using the Dual-Luciferase Reporter Assay System (Promega, Madison, WI, USA). Firefly luciferase activity was normalized to that of Renilla luciferase.

### Flow cytometry analysis of neuronal markers

On day 1 after the second step of induction, the ADMSCs were subjected to flow cytometry to quantify the proportion of cells with positive neurogenic markers. The cells were fixed using 4% paraformaldehyde at room temperature for 30 min and permeabilized using 0.1% TritonX-100+2% BSA in PBS at 37°C for another 30 min. Then the cells were incubated with primary anti-NSE antibody (1:100, ab53025, Abcam) for one hour, anti-GFAP (1:100, ab10062, Abcam) for 30 min or anti-Tuj-1 (1:100, ab18207, Abcam) for 30 min. For anti NSE and anti-Tuj-1, the secondary antibody used was Alexa Fluor^®^ 488 goat anti-rabbit IgG (H&L) (1:4000, ab150077, Abcam) for 30 min at 22°C. For anti-GFAP, the secondary antibody used was DyLight^®^ 488 goat anti-mouse IgG (H+L) (1:500, ab96879, Abcam) for 30 min at 22°C. The proportion of cells with active neuronal markers were then analyzed using a FACSCaliber (BD Biosciences, Franklin Lakes, NJ, USA). Data acquisition was done using CellQuest 3.2 software (BD Biosciences). Each test was performed with at least three repeats.

### Intracellular Calcium Measurements

To measure intracellular calcium concentration, cells were incubated with 2 μM Fura-2-AM in Krebs-Ringer solution buffered with HEPES (125 mM NaCI, 5 mM KCI, 1.2 mM MgSO_4_, 2 mM CaCl_2_, 10 mM glucose, and 25 mM HEPES/NaOH, pH 7.4) for 40 min at 37°C. Then the cells were washed twice with pre-warmed Krebs-Ringer solution and transferred to the recording chamber of an inverted microscope (Axiovert 100, Zeiss, Germany) equipped with a Ca^2+^ imaging unit. Polychrome IV (TILL Photonics, Germany) was used as a light source. Fura-2 fluorescence images were collected with a PCO Super VGA SensiCam (Axon Instruments, CA, USA) and analyzed with Axon Imaging Workbench 6.2 software (Axon Instruments). Intracellular calcium dynamics of the cells after exposure to 50 nM KCI, 1 mM ATP or field electrical stimulation (40 action potentials at 20 Hz) was recorded. Single cell 340/380 nm fluorescence ratios, acquired at 1-4/s, were analyzed with an Origin 6.0 software (Microcal Software Inc., MA, USA).

### Electrophysiological Recordings

Whole-cell patch-clamp recordings were performed on ADMSCs, differentiated ADMSCs (diff. ADMSCs). Rat hippocampal neurons were used as positive control. Patch pipettes (2-4MΩ) were pulled using a micropipette electrode puller (Sutter Instruments) and filled with intracellular solution (130mM K-gluconate, 10mM KCI, 1mM EGTA, 10mM HEPES-NaOH, 2mM MgCl_2_, 4mM MgATP, and 0.3mM Tris-GTP). Cells plated on glass cover slips were placed in a recording chamber perfused continuously with extracellular solution (125mM NaCI, 5mM KCI, 1.2mM MgSO_4_, 1.2 KH_2_PO_4_, 2mM CaCl_2_, 6mM glucose, and 25 mM HEPES-NaOH, pH = 7.4). Membrane potentials were recorded under the *I* = 0 configuration mode. Signals were recorded using Multiclamp 700B amplifiers and digitized with Digidata 1440 (Axon Instruments). Signals were amplified, sampled at 10 kHz, and filtered to 2 or 5 kHz.

### Statistical analysis

Data were presented in the form of means ± standard deviation (SD) based at least three repeats of three times independent studies. One-way ANOVA was performed to compare means of in multiple group experiments. Comparison between groups was performed using the unpaired t test. A two-sided P value of <0.05 was considered statistically significant.

## Results

### MiR-124 is significantly upregulated during neurogenic transdifferentiation of ADMSCs

**Fig 1:**
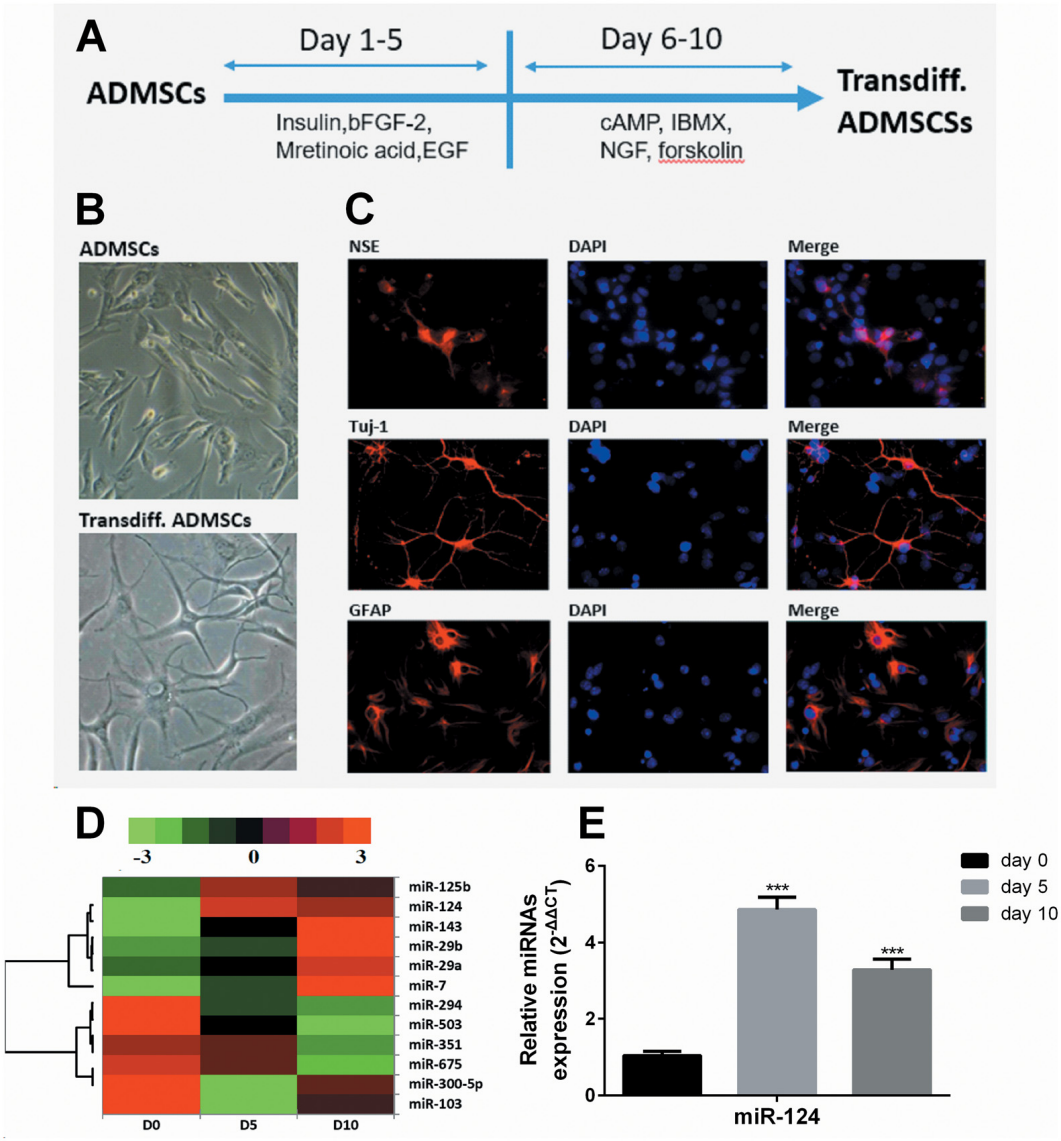
MiR-124 is significantly upregulated during neurogenic transdifferentiation of ADMSCs. (A) A protocol outlined describing the strategy to induce neurogenic transdifferentiation of ADMSCs. (B) Representative morphological images of ADMSCs before induction (up) and on day 1 post-induction (down). (C) Representative immunofluorescent images of neuronal markers (NSE and Tuj-1) and glial cell marker (GFAP) expressed in differentiated ADMSCs on day 1 post-induction. Red: neurogenic markers. Blue: DAPI. (D) A total of 12 differential expressed miRNAs, including 6 up-regulated and 6 down-regulated, were identified in the ADMSCs on day 0, 5 and 10 of the induction. The criteria was fold change ≥3 from day 0 to day 10, *p*<0.05. Columns represent samples and rows represent miRNAs (black, green, and red correspond to unchanged, down-regulated and upregulated, respectively). D0: day 0, D5: day 5, D10: day 10. (E) QRT-PCR analysis of miR-124 expression of the ADMSCs on day 0, day 5 and day 10 of induction. Transdiff. = transdifferentiated. * *p*<0.05, ** *p*<0.01, *** *p*<0.001.

To induce neurogenic transdifferentiation, ADMSCs were induced using a protocol showed in Fig 1A. After the two steps induction, the transdifferentiated (trandiff.) ADMSCs presented neuronal-like morphology (Fig 1B). The transdifferentiation was also visualized by immunofluorescent staining of neurogenic markers, including NSE, Tuj-1 and GFAP (Fig 1C). By performing miRNAs microarray, we found a series of miRNAs were significantly changed during the induction process. The six most upregulated and most downregulated miRNAs are shown in Fig 1D. QRT-PCR analysis also showed that miR-124 expression is consistently increased during the induction (Fig 1E). In fact, previous studies reported that miR-124 is an important miRNA modulating neuronal and astrocyte differentiation [19, 20] and may also regulate neuronal transdifferentiation of MSCs [7, 21]. Therefore, we decided to explore more details of its downstream regulation.

### MiR-124 knockdown hampers neurogenic transdifferentiation of ADMSCs

**Fig 2:**
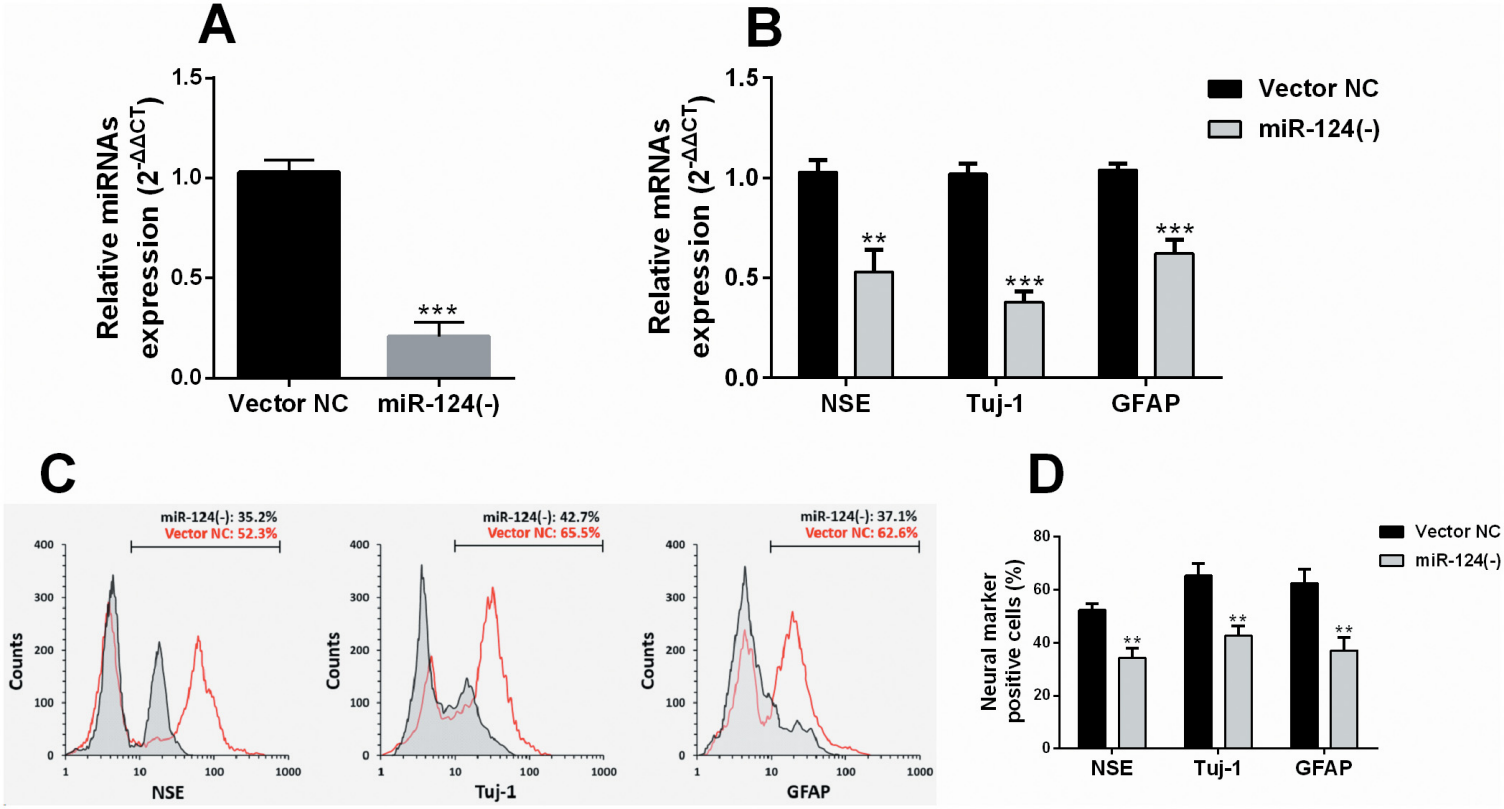
MiR-124 deficiency can affect neurogenic transdifferentiation of ADMSCs. (A) QRT-PCR analysis of miR-124 expression in ADMSCs infected with the pLV-miR-124 locker lentiviral particles or the vector negative control (Vector NC). (B) QRT-PCR analysis of NSE, Tuj-1 and GFAP mRNA expression in ADMSCs (with or without miR-124 knockdown) on day 1 post-induction. (C) Representative images of flow cytometry analysis of ADMSCs (with or without miR-124 knockdown) with positive neurogenic markers on day 1 post-induction. (D) Quantification of the proportion of ADMSCs with positive neurogenic markers showed in (C). * *p*<0.05, ** *p*<0.01, *** *p*<0.001.

To further verify the effect of miR-124 on neurogenic transdifferentiation of ADMSCs, the ADMSCs were firstly transfected with pLV-miR-124 locker particles or the vector negative control (Vector NC) (Fig 2A). MiR-124 knockdown resulted in substantially decreased expression of NSE, Tuj-1 and GFAP mRNA after induction (Fig 2B). In addition, miR-124 knockdown also reduced the proportion of cells with positive NSE (Vector NC vs. miR-124(-): 53.3±2.5% vs. 34.7±3.7%), Tuj-1 (Vector NC vs. miR-124(-): 64.9±4.7% vs. 42.8±3.6%) and GFAP (Vector NC vs. miR-124(-): 62.8±5.2% vs. 37.4±4.8%) after induction (Fig 2C and 2D), suggesting a repressed transdifferentiation.

### MiR-124 knockdown hampers the acquired electrophysiological properties due to transdifferentiation

**Fig 3:**
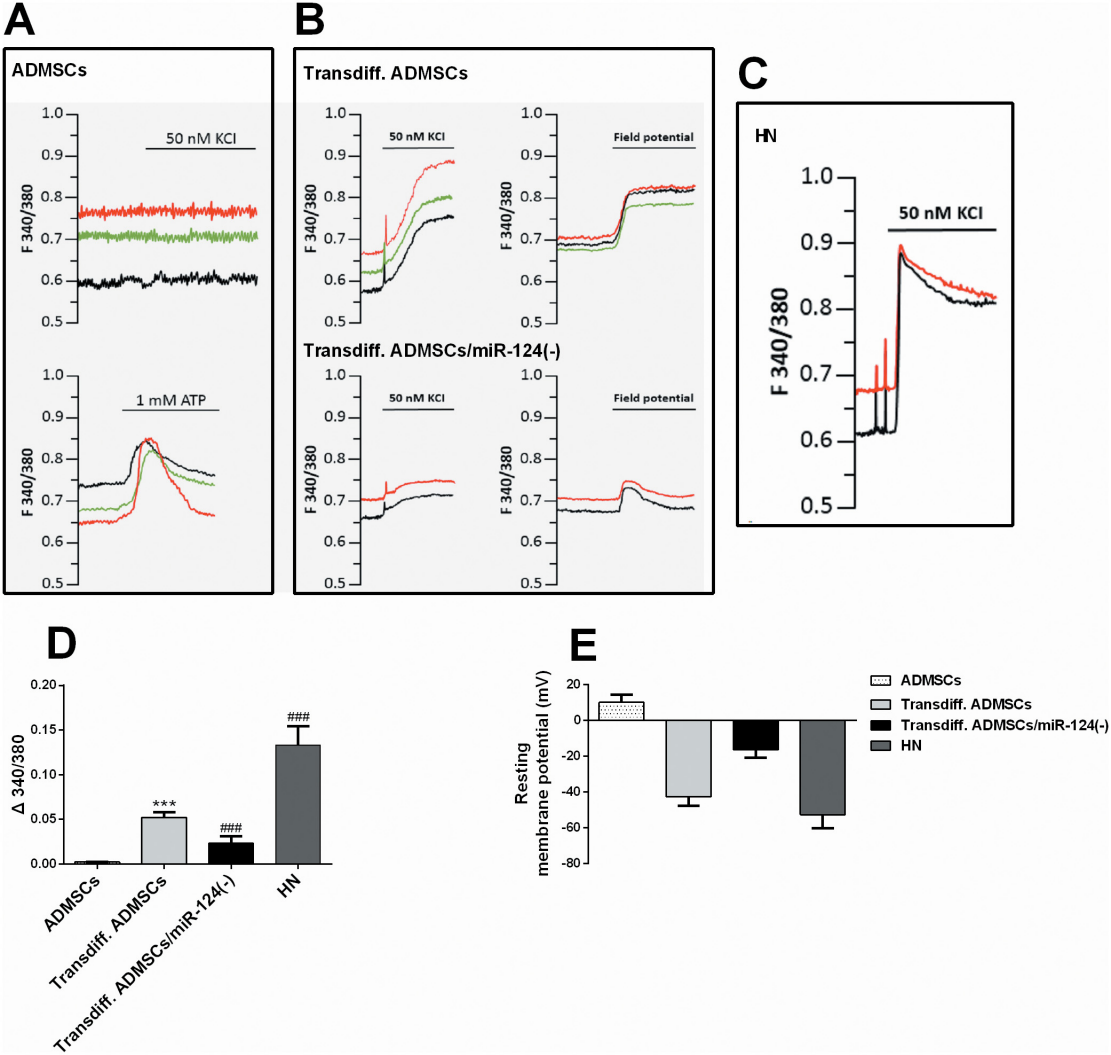
MiR-124 knockdown hampers the acquired electrophysiological properties due to transdifferentiation. (A) Representative traces of intracellular calcium dynamics (ratiometric acquisition) showing lack of response of untransdifferentiated ADMSCs at biochemical depolarization with 50 nM KCI while still preserving their functional response following exposure to 1 mM ATP. (B) Representative traces of intracellular calcium dynamics (ratiometric acquisition) showing functional response of transdiff. ADMSCs (without miR-124 knockdown: up; with miR-124 knockdown: down) both at biochemical depolarization with KCI as well as the electrical field potential stimulation. However, the responses of the transdiff. ADMSCs/miR-124(-) were significantly weaker. (C) Representative traces of intracellular calcium dynamics (ratiometric acquisition) of mature hippocampal neurons (HN) at biochemical depolarization with 50 nM KCI. (D) Quantification of the peak of intensity of response of transdiff. ADMSC and neurons following exposure to 50 mM KCI (Δ340/380; ADMSCs naive = 0.002±0.001, n = 6; transdiff. ADMSCs = 0.052±0.006, n = 6; transdiff. ADMSCs/miR-124(-) = 0.023±0.008, n = 6; HN = 0.133±0.021, n = 6). (E) Quantitative evaluation of membrane potential values in ADMSCs, transdiff. ADMSCs (with or without miR-124 knockdown) and in hippocampal neurons (ADMSCs naive: 10.13±4.22 mV, n = 6; transdiff. ADMSCs = -42.52±5.31 mV, n = 6; transdiff. ADMSCs/miR-124(-) = -16.37±4.42 mV, n = 6; HN = -52.75±7.48, n = 6). *: comparison with ADMSCs; #: comparison with transdiff. ADMSCs. * and # *p*<0.05, ** and ## *p*<0.01, *** and ### *p*<0.001.

In order to check whether the induced neuronal-like cells have neuronal-like electrophysiological properties and how miR-124 could affect the properties, we analyzed the intracellular calcium responses of the transdifferentiated (transdiff.) ADMSCs (with or without miR-124 knockdown) to depolarizing stimuli by ratiometric imaging. Undifferentiated ADMSCs did not show any calcium response when exposed to 50 mM KCI, but showed calcium transients in response to 1mM ATP stimulation (Fig 3A). However, the transdiff. ADMSCs responded to calcium influx to either 50 mM KCI or field electrical stimulation (40 action potentials at 20 Hz) (Fig 3B). These results suggest that the transdiff. ADMSCs acquired membrane excitability properties resemble to mature neuron cells (Fig 3C). However, unlike the primary neurons showed a sudden calcium transient followed by a slow recovery, transdiff. ADMSCs showed a slower calcium increase and a sustained calcium plateau (Fig 3B and 3C). These results suggested the neuronal-like transdiff. ADMSCs are still immature. Compared with normal transdiff. ADMSCs, the response of transdiff. ADMSCs/miR-124(-) to either 50 mM KCI or field electrical stimulation was weaker (Fig 3B and 3D). The acquisition of neuronal phenotype during transdifferentiation was further assessed using whole cell patch-clamp recording. The patched transdiff. ADMSCs showed a significant reduction of membrane potential values, which were similar to the mature neurons. However, although the transdiff. ADMSCs/miR-124(-) also showed a significant reduction of membrane potential, the level of reduction was much lower (Fig 3E). These results suggest that miR-124 knockdown hampers the acquired electrophysiological properties due to transdifferentiation

### MiR-124 can directly target RHOA mRNA and regulate its expression

**Fig 4:**
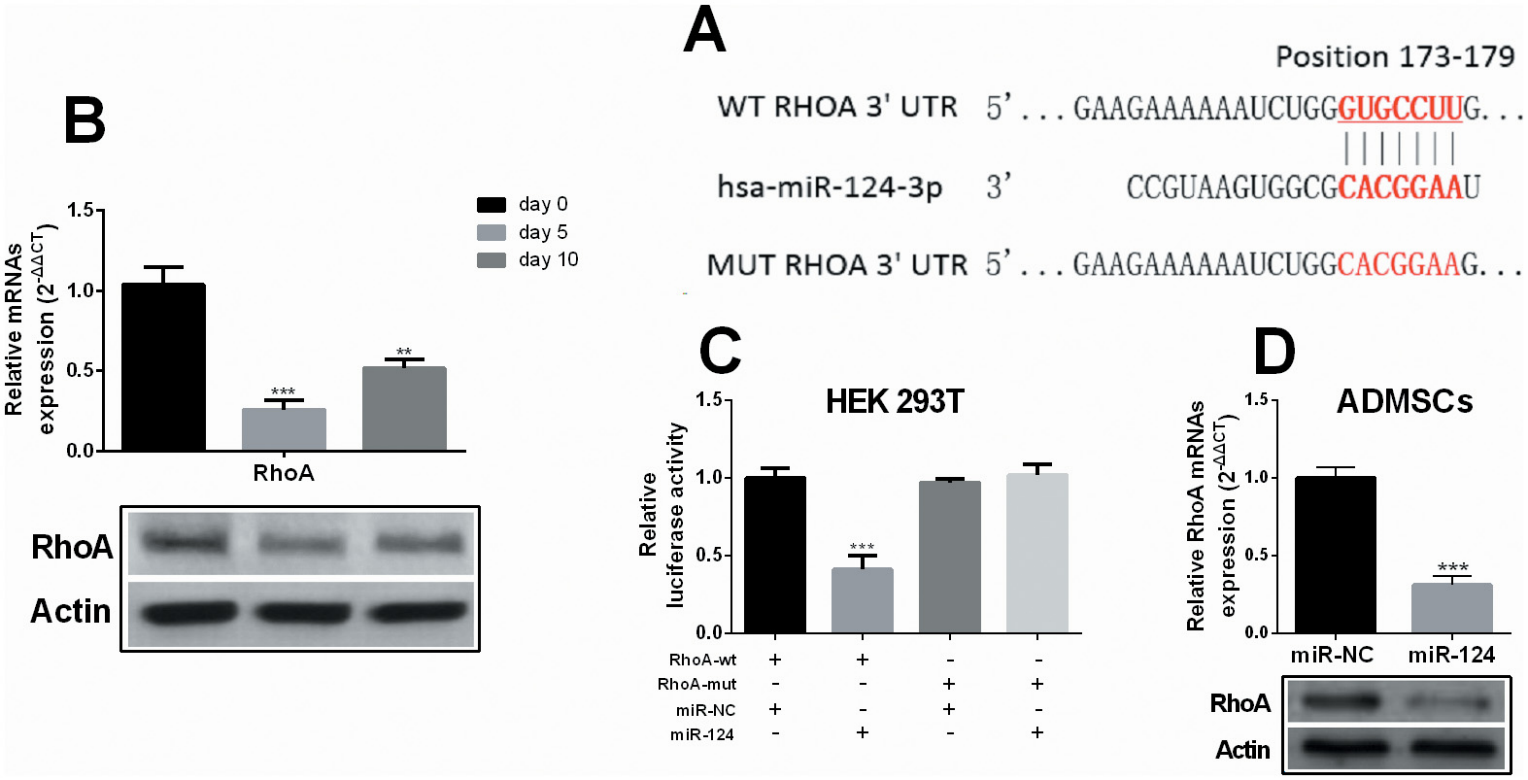
MiR-124 can directly target RHOA mRNA and regulate its expression. (A) QRT-PCR (up) and western blot (down) analysis of RhoA expression in ADMSCs on day 0, 5 and 10 during the transdifferentiation induction. (B) Predicted binding site between 3’UTR of RhoA mRNA and miR-124 and the designed mutant sequence. (C) Dual luciferase assay of relative luciferase activity of pGL3-RhoA-WT (RhoA-WT) and pGL3-RhoA-MUT (RhoA-WT) transfected with 50 nM miR-124 mimics or the negative control in HEK 293T cells. Firefly luciferase activity was normalized to that of Renilla luciferase. (D) QRT-PCR (up) and western blot (down) analysis of RhoA expression in ADMSCs infected with the pLV-miR-124 locker lentiviral particles or the negative control. * *p*<0.05, ** *p*<0.01, *** *p*<0.001.

Previous studies found that miR-124 can regulate neuronal transdifferentiation by targeting SP1 mRNA[7] or Ezh2 mRNA [19]. However, one miRNA usually targets multiple genes and might be involved in a wide regulative network. Therefore, we further investigated whether other genes are involved in miR-124’s regulation of neuronal transdifferentiation of ADMSCs. Based on our preliminary studies, we found RhoA has a putative binding site with miR-124 (Fig 4A). By quantifying RhoA expression in the ADMSCs during the induction process, it was observed that RhoA expression was significantly downregulated at both mRNA and protein level (Fig 4B). Therefore, we performed the dual luciferase assay to verify the putative binding. MiR-124 mimics can only repress the luciferase activity of the reporter carrying wild type RhoA 3’UTR sequence, but not the reporter with the mutant sequence in HEK 293T cells (Fig 4C). In ADMSCs, infection with pLV-miR-124 expression particles also significantly decreased RhoA expression at both mRNA and protein level (Fig 4D). These results suggest that miR-124 can directly target RhoA and regulate its expression.

### MiR-124 can affect neurogenic transdifferentiation of ADMSCs partly through RhoA/ROCK1 signaling pathway

**Fig 5:**
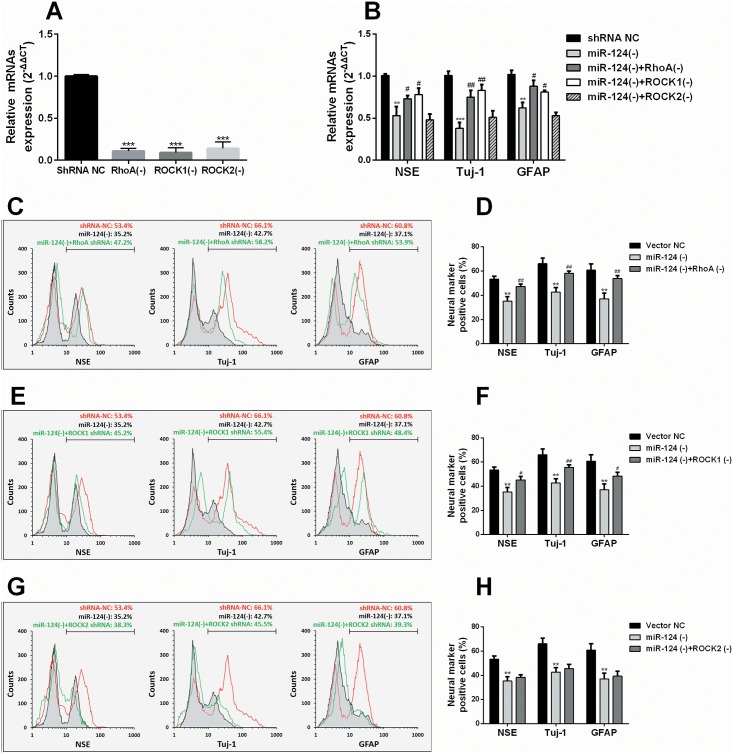
MiR-124 enhances neuronal transdifferentiation of ADMSCs partly through RhoA/ROCK1 signaling pathway. (A) QRT-PCR analysis of RhoA, ROCK1 and ROCK2 mRNA expression in ADMSCs infected with the RhoA shRNA lentiviral particles, ROCK1 shRNA lentiviral particles, ROCK2 shRNA lentiviral particles or the negative control. (B) QRT-PCR analysis of NSE, Tuj-1 and GFAP mRNA expression in ADMSCs infected with the pLV-miR-124 locker lentiviral particles, co-infected with the pLV-miR-124 locker lentiviral particles and the RhoA shRNA lentiviral particles, ROCK1 shRNA lentiviral particles or ROCK2 shRNA lentiviral particles on day 1 after transdifferentiation induction. (C, E and G) Representative images of flow cytometry analysis of neurogenic markers (NSE, Tuj-1 and GFAP) positive ADMSCs infected with the pLV-miR-124 locker lentiviral particles and co-infected with the pLV-miR-124 locker lentiviral particles and the RhoA shRNA lentiviral particles (C), ROCK1 shRNA lentiviral particles (E) or ROCK2 shRNA lentiviral particles (G) on day 1 after transdifferentiation induction. (D, F and H) Quantification of the proportion of ADMSCs with positive neurogenic markers showed in C, E and G. *: comparison with shRNA NC or vector NC; #: comparison with transdiff. ADMSCs/miR-124(-). * and # *p*<0.05, ** and ## *p*<0.01, *** and ### *p*<0.001.

**Fig 6:**
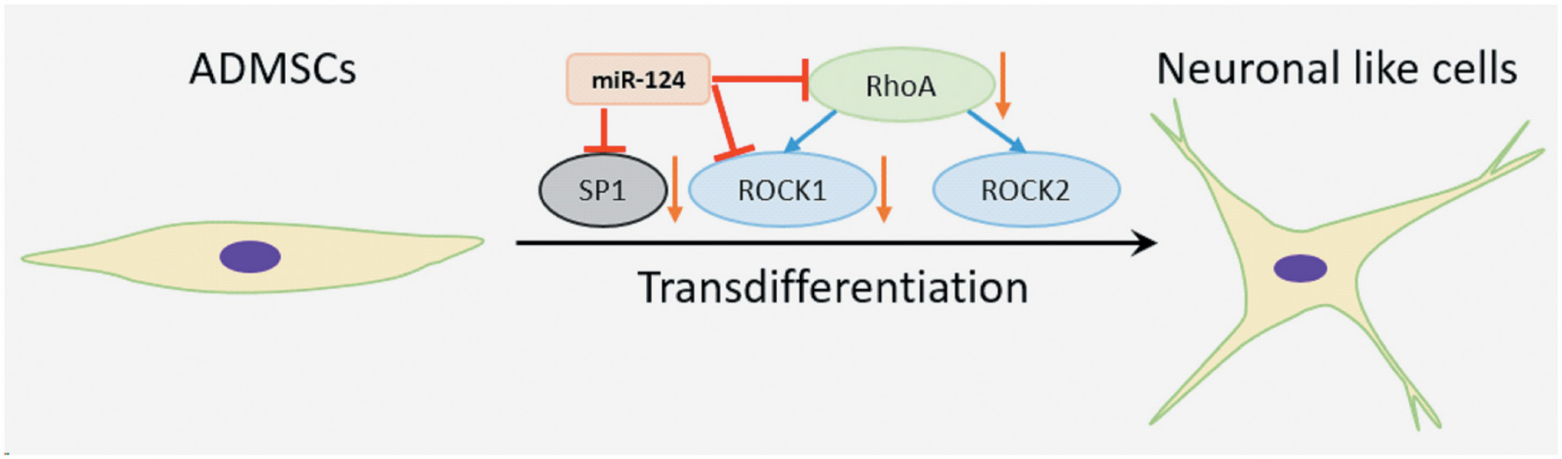
The schematic diagram of miR-124’s regulation over neurogenic transdifferentiation of ADMSCs.

To further explore whether RhoA/ROCK signal is involved in miR-124’s regulation over neurogenic transdifferentiation of ADMSCs, ADMSCs were firstly transfected with RhoA, ROCK1 or ROCK2 shRNA lentiviral particles before induction (Fig 5A). Knockdown of endogenous RhoA or ROCK1, but not ROCK2 partly restored the expression of neuronal markers hampered by miR-124 shRNA (Fig 5B). Flow cytometry analysis showed that RhoA and ROCK1 shRNA, but not ROCK2 shRNA partly reversed the effect of miR-124 shRNA on repressing the proportion of transdiff. ADMSCs with positive neuronal markers (Fig 5C–5H). These results suggest that RhoA or ROCK1 shRNA rescued neurogenic transdifferentiation hampered by miR-124 knockdown. In combination with the findings reported in previous studies [7, 14, 15], it is possible that miR-124 can modulate neurogenic transdifferentiation of ADMSCs by simultaneously repressing SP1, RhoA and ROCK1 (Fig 6).

## Discussion

A wide range of studies reported that MSCs have the ability to transdifferentiate into mature neuron-like cells that present multiple neuronal properties, such as synaptic transmission, action potential, secretion of dopamine and neurotrophic factors, and spontaneous postsynaptic current [22–24]. Therefore, MSCs might have potential therapeutic applications in the treatment of neurological diseases. In this study, we verified that the ADMSCs can be induced to neuronal-like cells by a two-step protocol. In addition, during the induction process, we also confirmed significantly upregulated expression of miR-124.

Previous studies reported that miR-124 may have an important role in neuronal differentiation [19, 20]. Ectopic expression of miR-124 in mouse neurogenic stem cells induced morphological changes and marker expressions consistent with neuronal differentiation [11]. Transfection of miR-124 and two transcription factors (MYT1L and BRN2) in combination can directly reprogram postnatal and adult human primary dermal fibroblasts to functional neurons under precisely defined conditions [25]. Another study also reported that miR-124 in combination with miR-9*, can instruct compositional changes of SWI/SNF-like BAF chromatin-remodeling complexes, a process important for neuronal differentiation and function, thereby contributing to neuronal fates [26]. Therefore, miR-124 might play a critical role in maintaining neuronal state of the cells. In the current study, knockdown of endogenous miR-124 hampered the transdifferentiation potential of ADMSCs. Although the cells with miR-124 knockdown still have the potential of transdifferentiation, the electrophysiological features of the differentiated cells were significantly weaker. This finding is consistent with one recent study that reported overexpression of miR-124 can promote neuronal transdifferentiation of bone marrow-derived MSCs [21]. However, how miR-124 modulates the differentiation process has not been fully revealed.

One recent study reported that miR-124 can directly target the 3'-UTR of Sp1 mRNA and decrease its expression, which in turn affects the neuronal transdifferentiation of ADMSCs [7]. Since miR-124 might play an important role in neuronal-like transdifferentiation, it is necessary to further explore its downstream regulations. Interestingly, another recent study found miR-124 can directly target and repress ROCK1 expression in M17 cells, through which it regulates neurite outgrowth and elongation [27]. Actually, the RhoA and its downstream signaling pathways might be quite critical for neuronal-like transdifferentiation of MSCs. Y-27632, a ROCK inhibitor can enhance the efficacy of bone marrow-derived MSCs transdifferentiation into neurons and neuroglial cells [14, 15]. SCIRR39, a gene usually upregulated in primary neuron injury and/or regeneration process, can increase the neurite extension in NGF-treated PC12 cells via RhoA [28]. Cyclic tensile loading, a mechanic methods used to promote neuronal transdifferentiation of MSCs is also partly through the regulation of Rho GTPases activity [16]. Our preliminary study observed that miR-124 has a putative binding site with 3’UTR of RhoA mRNA. Therefore, we hypothesized that miR-124 can modulate neuronal-like transdifferentiation of ADMSCs through repressing RhoA. By performing dual luciferase assay and western blot analysis, we verified this binding site. Since RhoA has two downstream effectors, ROCK1 and ROCK2, we further investigated which one is involved in MSCs transdifferentiation. Knockdown of endogenous ROCK1, but not ROCK2, partly rescued neuronal transdifferentiation hampered by knockdown of miR-124. Therefore, we confirmed that the miR-124/RhoA/ROCK1 is an important axis involved in neuronal-like transdifferentiation of ADMSCs. In fact, ROCK1 is an important downstream effector of RhoA modulating cell fate. For example, miR-125a-3p and miR-483-5p can promote adipogenesis of ADMSCs via suppressing the RhoA/ROCK1/ERK1/2 pathway [29]. MiR-335-5p can promote chondrogenesis of mouse MSCs by downregulating Daam1 and ROCK1 and increasing SOX9 [30]. In addition, it is possible that the role of RhoA/ROCK1 signaling pathway is related to inducing environment and might be interacted with other signaling pathways. In the future, it is necessary to carry out more detailed research to explore its interaction with other transdifferentiation related factors.

## Conclusion

In this study, we showed that miR-124 is an important miRNA modulating neuronal-like transdifferentiation of ADMSCs. We also demonstrated that the miR-124/RhoA/ROCK1 is an important signaling pathway in the transdifferentiation. These findings provided some fundamental information for future use of ADMSCs as an agent for regenerative medicine and cell therapy for neurological diseases.

## Supporting Information

S1 Table Primer Sequences for qRT-PCR. (DOCX)
