# Supplementary material for: MiR-124 Promote Neurogenic Transdifferentiation of Adipose Derived Mesenchymal Stromal Cells Partly through RhoA/ROCK1, but Not ROCK2 Signaling Pathway
Source: PLoS One. 2016 Jan 8;11(1):e0146646. doi: 10.1371/journal.pone.0146646 (PMC4706435; doi:10.1371/journal.pone.0146646)
Supplement: S1 Table — (DOCX) [file pone.0146646.s001.docx]

**S1 Table. Primer Sequences for qRT-PCR**

| **Gene** | **Primer Sequences** |
| --- | --- |
| RhoA | F: 5’-CGGGAGCTAGCCAAGATGAAG-3’  R: 5’-CCTTGCAGAGCAGCTCTCGTA-3′ |
| NSE | F: 5’-TATGGATGTGGCTGCCTCTG-3’  R: 5’-TGGTGATTGGTATGGATGTGG-3′ |
| GFAP | F: 5’-ATCGAGATCGCCACCTACAG-3’  R: 5’-CTCACATCACCACGTCCTTG-3′ |
| Tuj-1 | F: 5’- AGCAAGAACAGCAGCTACTTCGT-3’  R: 5’- GATGAAGGTGGAGGACATCTTGA-3′ |
